# Controlled Delivery Formulations

**DOI:** 10.3390/pharmaceutics13030374

**Published:** 2021-03-12

**Authors:** Marta Gonzalez-Alvarez

**Affiliations:** Department of Engineering, Pharmaceutical Technology Area, Miguel Hernandez University, Elche. San Juan Campus, 03550 Alicante, Spain; marta.gonzalez@umh.es

In the last few decades, controlled release formulations have gained an extraordinary interest. Controlled release formulations are designed and obtained to release active drugs with a specific release rate and at specific sites in order to increase therapeutic effects and benefits.

There are different types of classifications for different controlled release formulations, which consider several factors such as the extended release, delayed time, or the strategic release process based on the physiological characteristics in the different administration routes. 

This Special Issue has included some of the most interesting current controlled delivery formulations and allows us to demonstrate the large number of applications and advantages. It contains twelve papers: ten original research papers and two reviews—articles have been included. 

The special volume includes three publications in which mesoporous silica particles have been used as a vehicle to reach a drug controlled release and a review about the possibilities of mesoporous materials in cancer treatment [1,2,3,4]. The publication by Harun et al. [1] focused on the design, synthesis and characterization of a mesoporous formulation in order to improve the biopharmaceutical properties of the ruthenium polypyridyl complex [Ru(dppz)2PIP]2+ (dppz: dipyridophenazine, PIP: (2-(phenyl)-imidazo[4,5-f][1,10]phenanthroline). This complex exhibits an important nuclease activity but its poor solubility limits its clinical applications. Results indicate that complex encapsulation in mesoporous silica nanoparticles reduces the IC50 value in the assayed cancerous cell lines, produces an increase in G1/S phase populations in all three cell lines and allows a controlled release of the drug by diffusion. 

The work entitled “pH-Dependent Molecular Gate Mesoporous Microparticles for Biological Control of Giardia intestinalis” published by my research group in collaboration with Dr. Martinez-Mañez’s group describes a new formulation of metronidazol encapsulated in mesoporous microparticles but, in this case, gated with a pH dependent molecular gate [2]. The new formulation allows drug delivery in duodenum, specifically reaching high drug concentrations in the place where a specific parasite is located. This strategy allows us to reduce the dose of drug administered and to avoid the side effects derived of the metronidazol sistemic administration due to its high bioavailavility.

The third publication, provided by the Dr. Martinez-Mañez’s group [3], describes a silica xerogel prepared from a low-cost precursor used as a drug carrier of linezolid. Linezolid is an antibiotic effective against gram+ infections. The mesoporous carrier offers the advantages of its structure, suitable pore and particle size and its ultralow density and the new nanoformulation show a synergic activity and it is active against gram—bacterias such as Escherichia coli, Pseudomonas aeruginosa and Staphylococcus aureus. Obtained results indicate that silica xerogels can be useful as antibacterial carriers and activity enhancers due to they can improve the antibacterial spectrum of action. This approach might be a powerful tool to overcome antibiotic resistance.

The review entitled “Progress in Mesoporous Silica Nanoparticles as Drug Delivery Agents for Cancer Treatment” exhaustively revises the different synthetic methods for mesoporous nanoparticles, their possibilities on surface functionalization, the passive and active targeting that allow the specific and controlled drug delivery, the exploration of the molecular gates in order to reach a stimulus responsive drug delivery, the possibility to incorporate a single stimulus responsive mechanism, or dual, or even multiple stimulus response mechanisms. The review concludes the great potential of mesoporous silica nanoparticles in diagnostic and treatment of cancer only limited by the knowledge of the physiological processes underlying this pathology [4].

Cancer treatment is a hot topic for new formulations in order to reach specificity and a reduction of side effects. The paper published by Saimi et al. [5] shows a very original carrier, the niosomes aerosolized, for the lung cancer treatment. The formulation Gemcitabine and Cisplatin was prepared in a simple way and seems to be reproducible and suitable for clinical translation. Release assays, in vitro toxicity results using cancerous lung cells as well as aerosol output indicate that the new formulation is a promising real option for lung cancer treatment although it needs further research work. The Special Issue includes an original proposal of Jagusiak et al. [6] in which controlled release of doxorubicin is reached from a new formulation composed of single-walled carbon nanotubes and congo red. This triple complex formed by single-walled carbon nanotubes, Congo red, and doxorubicin is a new promising drug delivery system due to its high loading capacity and pH dependent release that ensure a slow controlled release of the drug in a specific location.

The paper “The Correlation between Physical Crosslinking and Water-Soluble Drug Release from Chitosan-Based Microparticles” published by Szymańska et al. [7] provides insights about microparticles obtained with chitosan glutamate and beta-glycerophosphate as an ion crosslinker using sprya-drying technique and containing water-soluble zidovudine. In vitro studies revealed that one of the proposals allows to obtain an optimal drug loading and release antirretroviral assays indicates a moderate burst effect followed with a prolonged drug release of up to 210 min.

The group led by Ariane van der Straten has focused the research work on developing a subcutaneous reservoir-style implant for long-acting delivery of tenofovir alafenamide (TAF) for HIV Pre-Exposure Prophylaxis [8]. They demonstrated the importance of choosing the proper excipient in order to reach a satisfactory release rate. The inclusion in the implant of the selected excipients (sesame oil, castor 543 oil) allows them to obtain sustained drug release during more than 240 days, as has been observed in the in vitro assays.

Other important fields of application of controlled release formulations can be focused in ophthalmological diseases. Alicia Lopez-Castellanos’s team explored glutathione formulations topical administration as antioxidant treatment for ocular diseases [9]. In this research work, a semisolid ocular insert using bioadhesive polymers with glycerol as a plasticizer and glutahion loaded were obtained using a solvent casting method. In vitro assays indicated that the new semisolid implant provided accurate dosing, improved pharmacokinetic parameter values, reduced administration frequency and diminished visual and systemic adverse effects and increased compliance compared with eyes drop solution formulation.

The article presented by Zlomke et al. [10] reveals interesting findings on using phospholipid extrudates implants for the controlled release of nicardipine. In this research work, the authors offer a proof-of concept and a basic in-vitro characterization about lipid implants based on a combination of saturated and unsaturated phospholipids in order to get a controlled nicardipine delivery system for the treatment of vasospasms in subarachnoid hemorrhage. The promising obtained formulation was found to be versatile, safe, able for specific release and very compatible for parenteral administration.

Eik and colleagues [11] explore the potential of new formulations prepared as extrudates but, in this case, with magnesium stearate and PLGA as the vehicle loaded with minocycline for peridontal applications. Peridontal diseases require the administration of antibiotics in controlled release formulations in order to optimize the therapy effectivity and safety. The antimicrobial activity of the new formulations manifested itself for twice as long as in commercial formulations indicating the potential for future peridontal infections.

Finally, 3D printing formulations are a new manufacturing approach with the capacity to revolutionize the future of pharmaceutical marked and this issue show a paper to illustrate its potential. Pereira et al. [12] carried out an extensive revision work about polymer selection for hot-melt extrusion coupled to fused deposition modelling in order to obtain optimal 3D printing formulations in pharmaceutics. This paper reviews the many strategies reported in the literature and systematizes the strategies to select the right polymer for additive manufacturing taking into account drug solubility, drug stability, the desired drug release profile, possible incompatibilities and therapeutic schedule.

This special volume highlights the large number of formulations and materials being investigated to achieve specific drug release profiles as well as the large number of potential therapeutic applications. It is necessary to invest in research in this field to ensure that these formulations can be developed and have options to reach the clinical application to improve the available therapeutic landscape.
